# Novel insights into the interplay between m^6^A modification and noncoding RNAs in cancer

**DOI:** 10.1186/s12943-020-01233-2

**Published:** 2020-08-07

**Authors:** You-Cai Yi, Xiao-Yu Chen, Jing Zhang, Jin-Shui Zhu

**Affiliations:** Department of Gastroenterology, Shanghai Jiao Tong University Affiliated Sixth People’s Hospital, No. 600 Yishan Road, Shanghai, 200233 China

**Keywords:** Noncoding RNAs, Cancer, m^6^A RNA methylation

## Abstract

N6-methyladenosine (m^6^A) is one of the most common RNA modifications in eukaryotes, mainly in messenger RNA (mRNA). Increasing evidence shows that m^6^A methylation modification acts an essential role in various physiological and pathological bioprocesses. Noncoding RNAs (ncRNAs), including miRNAs, lncRNAs and circRNAs, are known to participate in regulating cell differentiation, angiogenesis, immune response, inflammatory response and carcinogenesis. m^6^A regulators, such as METTL3, ALKBH5 and IGF2BP1 have been reported to execute a m^6^A-dependent modification of ncRNAs involved in carcinogenesis. Meanwhile, ncRNAs can target or modulate m^6^A regulators to influence cancer development. In this review, we provide an insight into the interplay between m^6^A modification and ncRNAs in cancer.

## Introduction

Up to now, more than 100 kinds of RNA modifications have been confirmed [1]. Among them, m^6^A RNA methylation is one of the most thoroughly studied modifications. m^6^A RNA modification occurs by methylation of the sixth N atom of adenine (A) in mRNAs or ncRNAs [2]. m^6^A modification sites tend to be found in the stop codons and 3′-Untranslated region (3′-UTR) of mRNA with a typical consensus sequence RRACH (R = G or A and H = A, C, or U) [3, 4]. Accumulating data show that m^6^A RNA methylation acts by modulating circadian rhythm, gene expression, cell differentiation, stress response, inflammatory response, and carcinogenesis [5–10]. According to the global cancer statistics, there were estimated 18.1 million new cases and 9.6 million deaths in 2018 [11]. Recent studies have shown that m^6^A modification acts a vital role in the diagnosis, treatment and prognosis of cancer patients as well as in carcinogenesis. It also regulates fly sex, virus genome, meiosis of yeast, tissue differentiation, germination, and collateral generation of Arabidopsis [12–15].

Noncoding RNAs (ncRNAs) including microRNAs (miRNAs), long non-coding RNAs (lncRNAs) and circular RNAs (circRNAs) act pivotal roles in cancer [16–18]. m^6^A modification can affect ncRNA splicing and maturation involved in carcinogenesis (Table 1). In this review, we summarize the latest progress about the interplay between m^6^A modification and ncRNAs in cancer.

**Table 1:** m^6^A methylation modifies ncRNAs in cancers

m^6^A component	Related non-coding RNA	Cancer	Function	Role in cancer	Regulation	References
METTL3	miR-25-3p	PDAC	Writers	Oncogene	Up-regulation	[45]
	miR-221、miR-222	Bladder cancer	Writers	Oncogene	Up-regulation	[46]
	miR-106b, miR-18a/b, miR-3607, miR-423, miR-30a, miR-320b/d/e	arsenite-induced carcinogenesis	Writers	Oncogene	Up-regulation	[47]
	miR-1246	CRC	Writers	Oncogene	Up-regulation	[49]
	miR-143-3p	Lung cancer	Writers	Oncogene	Up-regulation	[50]
METTL14	miR-126	HCC	Writers	Anti-oncogene	Down-regulation	[48]
METTL3	lncRNA FAM225A	NPC	Writers	Oncogene	Up-regulation	[67]
	lncRNA LINC00958	HCC	Writers	Oncogene	Up-regulation	[65]
	lncRNA RP11	CRC	Writers	Oncogene	Up-regulation	[68]
	MALAT1	NSCLC	Writers	Oncogene	Up-regulation	[69]
METTL14	XIST	CRC	Writers	Anti-oncogene	Down-regulation	[70]
METTL3/METTL14	LNCAROD	HNSCC	Writers	Oncogene	Up-regulation	[66]
ALKBH5	lncRNA NEAT1	GC	Erasers	Oncogene	Up-regulation	[71]
	lncRNA FOXM1-AS	glioblastoma	Erasers	Oncogene	Up-regulation	[57]
YTHDF1	LINC00278	ESCC	Readers	Anti-oncogene	Down-regulation	[72]
IGF2BP2	lncRNA DANCR	Pancreatic cancer	Readers	Oncogene	Up-regulation	[60]

*PDAC* pancreatic ductal adenocarcinoma, *HCC* hepatocellular cancer, *NPC* nasopharyngeal cancer, *GC* gastric cancer, *CRC* colorectal cancer, *NSCLC* non-small cell lung cancer, *HNSCC* head and neck squamous cell carcinoma, *ESCC* esophageal squamous cell carcinoma

## Molecular compositions of m^6^A RNA methylation

**Fig. 1:**
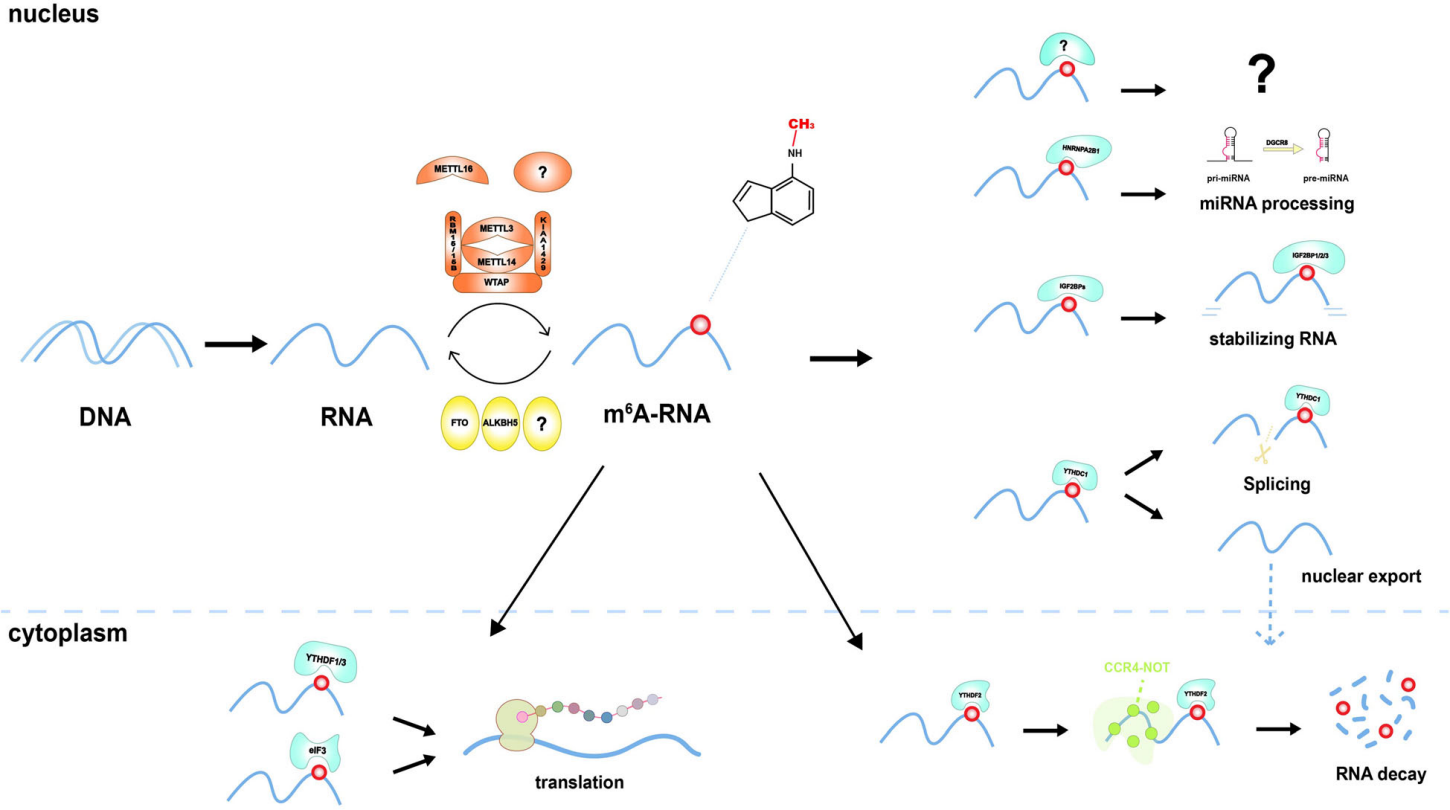
m6A modification is a dynamic and reversible process. m6A modification can be executed by “Writers” (METTL3/14, WTAP, KIAA1429, RBM15/15B, METTL16), demethylated by “Erasers” (FTO and ALKBH5) and regulated by “readers” (YTHDF1–3, YTHDC1–2, IGFBPs, eIF3 and HNRNPA2B1)

Molecular compositions of m^6^A RNA methylation include m^6^A methyltransferase, m^6^A demethylase, and m^6^A recognition factors (Fig. 1). m^6^A methyltransferases, called “writers” contain methyltransferase-like 3 (METTL3) [19], METTL14 [20], Wilms tumor 1-associated protein (WTAP) [2], KIAA1429 [21], METTL16 [22] and RNA-binding motif protein 15/15B (RBM15/15B) [23]. METTL3 regulates the circadian clock of hepatic lipid metabolism and hematopoiesis [24, 25]. METTL3/14 depletion promotes myeloid differentiation and suppresses the progression of acute myeloid leukemia (AML) [26, 27]. METTL16 maintains the levels of methyl donor S-adenosylmethionine (SAM) [28]. WTAP connects METTL3/14 to form a complex, anchored to the nucleus to catalyze m^6^A methyltransferase [2].

m^6^A methylation is dynamic and can be reversed by m^6^A demethylase, also named as m^6^A “erasers”, containing fat mass and obesity-associated protein (FTO) and alkB homologue 5 (ALKBH5) [29, 30]. FTO shares the motifs with Fe (II)- and 2-oxoglutarate-dependent oxygenase and is related to increased fat mass [31]. FTO harbors an efficient oxidative demethylation activity and reduces the m^6^A levels of mRNAs [30]. ALKBH5 is responsible for RNA splicing and stability and causes the degradation of abnormal transcripts in spermatocytes and round spermatids [32].

m^6^A recognition factors, known as “readers,” consist of YT521-B homology (YTH) domain family (YTHDF1/2/3) [33], YTH domain-containing proteins (YTHDC1/2) [12], heterogeneous nuclear ribonucleoprotein (HNRNP) protein families [33], eukaryotic translation initiation factor 3 (eIF3) [23], and insulin-like growth factor-2 mRNA-binding proteins 1/2/3 (IGF2BP1/2/3) [34]. m^6^A recognition factors act in oligodendrocyte progenitor cells and oligodendrocyte fate [35]. YTHDF1 controls pre-crossing axon guidance in the spinal cord by regulating m^6^A-modified Robo3.1 [36]. HNRNPA2B1 can initiate the immune response to DNA viruses by regulating interferon-α/β and stimulator of interferon genes (STING)-dependent antiviral signaling [37].

## m^6^A modification of miRNAs in cancer

As is known to us, the dysregulation of miRNAs is involved in various bio-behaviors, such as mouse prenatal development, immune response, inflammatory response and carcinogenesis [38–41]. METTL3 or HNRNPA2B1 facilitates pri-miRNA processing by recruiting RNA-binding protein DiGeorge syndrome critical region 8 (DGCR8) [42, 43]. METTL3 suppresses osteogenic processes by promoting the maturation of miR-7212-5p and downregulating its target fibroblast growth factor receptor 3 (FGFR3) [44].

### Tumor proliferation and tumorigenesis

**Fig. 2:**
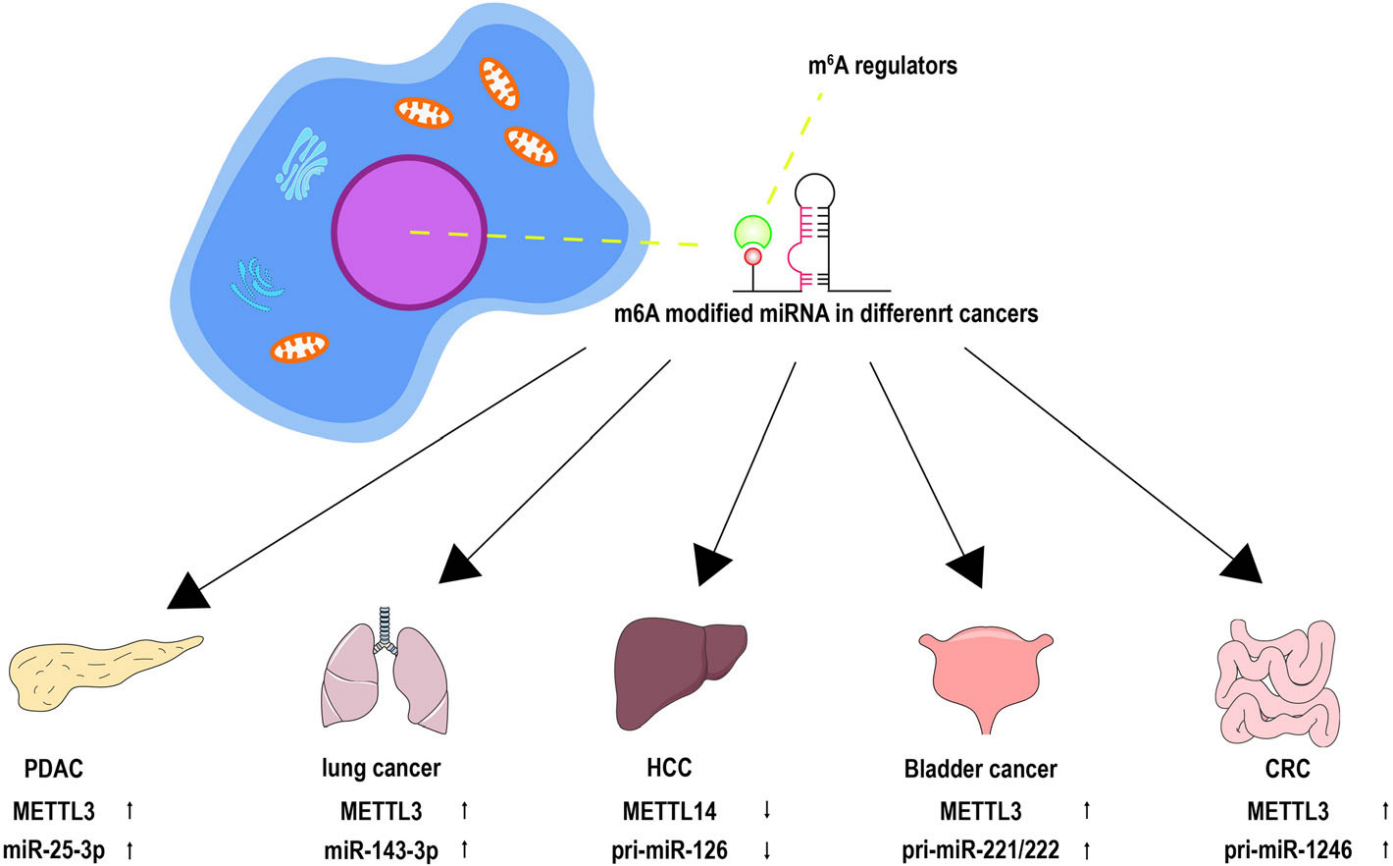
m6A methylation modifies miRNAs to regulate tumorigenesis and metastasis in multiple cancers including PDAC, lung cancer, HCC, bladder cancer, and CRC

m^6^A methylation can modify the maturation of miRNAs involved in cell proliferation and tumorigenesis (Fig. 2). miR-25-3p acts as a pivotal role in pancreatic ductal adenocarcinoma (PDAC). Cigarette smoke condensate (CSC) mediates METTL3 to promote miR-25-3p maturation in PDAC tumorigenesis [45]. METTL3 also enhances the binding of pri-miR-221/222 with DGCR8 involved in the proliferation of bladder cancer [46]. m^6^A modification affects arsenite-induced carcinogenesis via modifying multiple miRNAs (miR-106b, miR-18a/b, miR-3607, miR-423, miR-30a, miR-320b/d/e) [47].

### Tumor invasion and metastasis

METTL14 promotes the maturation of pri-miR-126 and suppresses the invasion and metastasis of hepatocellular carcinoma (HCC) [48]. METTL3 facilitates the maturation of pri-miR-1246 to enhance the metastasis of colorectal cancer (CRC) [49]. METTL3 also accelerates the maturation of miR-143-3p, leading to the formation of METTL3/miR-143-3p/vasohibin-1 axis to favor the metastasis of lung cancers [50].

## m^6^A modification of lncRNAs in cancer

**Fig. 3:**
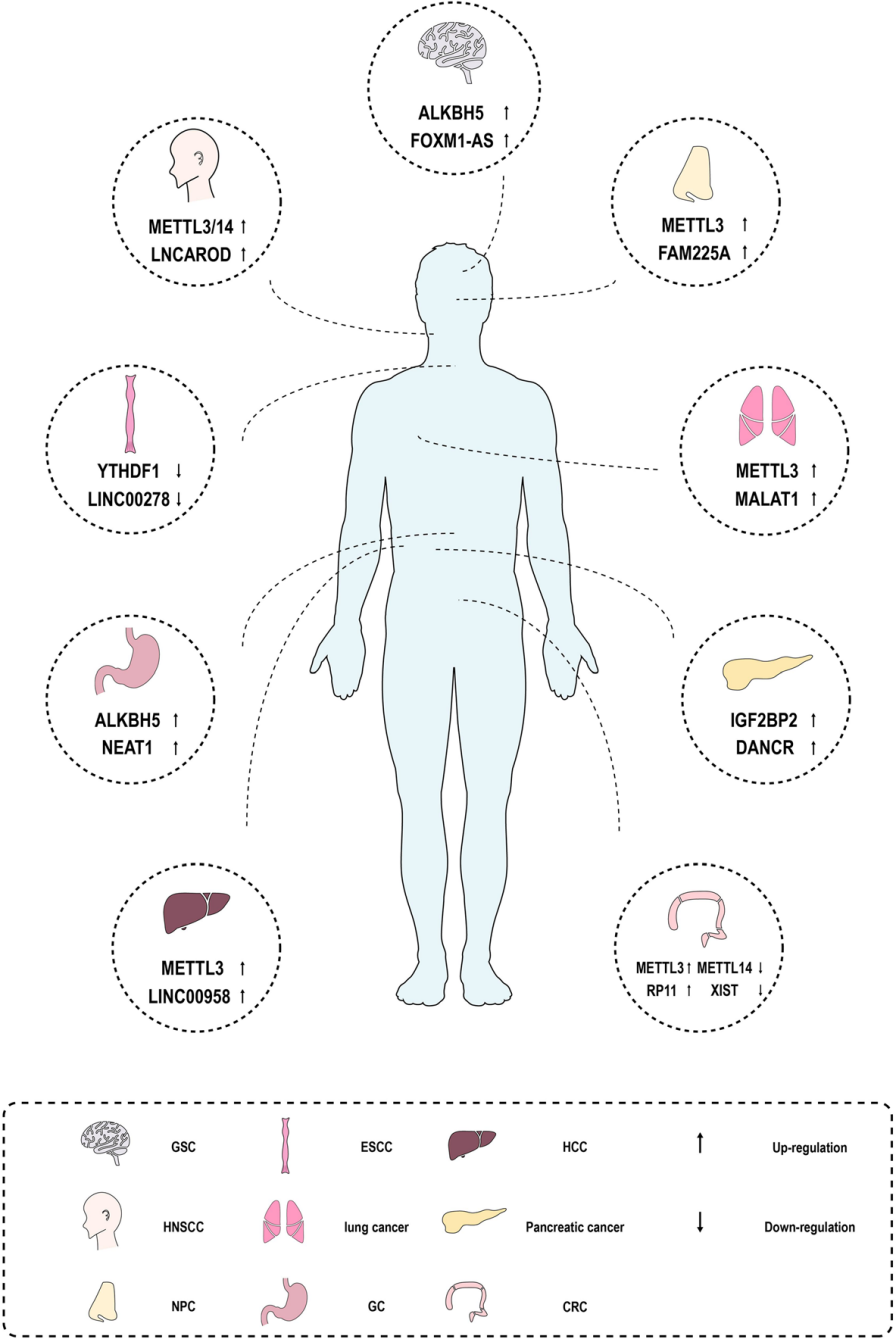
m6A methylation modifies lncRNAs to participate in tumorigenesis and metastasis in multiple cancers including GSC, HNSCC, NPC, ESCC, lung cancer, GC, HCC, pancreatic cancer and CRC

LncRNAs, a subgroup of non-coding RNAs over 200 nucleotides in length can be modified by m^6^A methylation in cancer (Fig. 3). m^6^A methylation facilitates lncRNA X-inactive specific transcript (XIST)-mediated transcriptional repression [51–53]. YTHDC1 preferentially recognizes the m^6^A residues of XIST and RBM15/15B and participates in XIST-mediated gene silencing [53]. However, RBM15/m^6^A-MTase complex is reported to act a minor role in XIST-mediated gene silencing [54]. YTHDF2 recognizes m^6^A methylation site of lnc-Dpf3 to promote its degradation and enhances the binding of lnc-Dpf3 with hypoxia-inducible factor 1-alpha (HIF-1α), leading to the suppression of the glycolysis and migration of dendritic cells [55]. METTL3 can modify metastasis-associated lung adenocarcinoma transcript 1 (MALAT1) to form the METTL3/MALAT1/miR-145/focal adhesion kinase (FAK) axis, contributing to the aggravation of renal fibrogenesis in obstructive nephropathy [56].

### Tumor proliferation and tumorigenesis

ALKBH5 has been found upregulated in glioblastoma and prompts the proliferation of glioblastoma stem-like cells (GSCs). A lncRNA antisense to forkhead box M1 (FOXM1-AS) promotes the interaction of ALKBH5 with forkhead box M1 (FOXM1) nascent transcripts to increase FOXM1 expression and GSCs tumorigenesis [57]. LncRNA Differentiation antagonizing non-protein coding RNA (DANCR) contributes to the tumorigenesis of multiple cancers [58, 59]. IGF2BP2 serves as an m^6^A reader to modify DANCR and favors the oncogenicity of pancreatic cancer [60]. MALAT1, the first lncRNA to be found associated with lung cancer, possesses a triple helix structure at its 3’end [61–63]. METTL16 interacts directly with MALAT1 triple helix and promotes cancer cell proliferation [64].

### Tumor invasion and metastasis

Long non-coding RNA 00958 (LINC00958) is upregulated by METTL3 and facilitates HCC cell migration and invasion by sponging miR-3619-5p [65]. METTL3/14 enhance the migration of head and neck squamous cell carcinoma (HNSCC) by upregulating lncRNA activating regulator of DKK1 (LNCAROD) [66]. METTL3-family with sequence similarity 225 member A (FAM225A)-integrin β3 (ITGB3)-FAK/PI3K/Akt axis facilitates the metastasis of nasopharyngeal cancer [67]. METTL3 mediates lncRNA RP11–138 J23.1 (RP11) or MALAT1-miR-1914-3p-Yes associated protein (YAP) axis to enhance the migration and invasion of CRC and non-small cell lung cancer (NSCLC) [68, 69]. METTL14 increases the m^6^A levels of XIST and suppresses the invasion of CRC [70]. ALKBH5 favors the invasion and metastasis of gastric cancer (GC) by demethylating lncRNA nuclear paraspeckle assembly transcript 1 (NEAT1) [71]. YTHDF1 restrains esophageal squamous cell carcinoma (ESCC) by interacting with long intergenic non-protein coding RNA 278 (LINC00278), but ALKBH5 harbors an opposite function [72].

## m^6^A modification of circRNAs in cancer

CircRNAs, a novel subset of ncRNAs generated by back-splicing, play a crucial role in protein translation [73]. METTL3 and YTHDC1 are associated with the metabolism of circular RNA zinc finger protein 609 (circ-ZNF609) and promote its production [74]. Minigenes of ribosomes-circRNAs (Ribo-circRNAs) can facilitate protein translation in drosophila heads and circ-ZNF609 boosts protein translation and myoblasts cell proliferation [75, 76]. m^6^A methylation has been reported to affect protein translation of cricRNAs [77, 78]. m^6^A motifs are enriched in circRNAs, and a single m^6^A site is regarded as a trigger to initiate the translation of circRNAs. m^6^A regulators METTL3/14, FTO, YTHDF3, and initiation factor eIF4G2 are involved in m^6^A-driven protein translation [78]. Mammalian cells can recognize the m^6^A modification on circRNAs to inhibit innate immunity by abrogating immune gene activation and adjuvant activity [79].

In addition, the dysregulation of circRNAs is associated with the progression of multiple cancers, such as breast cancer, gastric cancer (GC), gallbladder cancer and cervical cancer [80–83]. YTHDC1 interacts with circRNA NOP2/Sun RNA methyltransferase 2 (circNSUN2) to facilitate its cytoplasmic export, which leads to colorectal liver metastasis by forming a circNUSN2/IGF2BP2/high mobility group AT-hook 2 (HMGA2) RNA-protein ternary complex in the cytoplasm [84]. m^6^A modification can be involved in the progression of GC by regulating circRNA poliovirus receptor-related 3 (circPVRL3) [85].

## m^6^A regulators are regulated by ncRNAs in cancer

NcRNAs have the capabilities to affect m^6^A levels involved in multiple biological processes (Table 2). miRNAs can modulate the binding between METTL3 and its target mRNAs to participate in the reprogramming efficiency of mouse embryonic fibroblasts (MEFs) [86]. miR-149-3p inhibits adipogenesis lineage differentiation and potentiates osteogenic lineage differentiation by targeting FTO [87]. miR-1266 inhibits CRC progression by targeting FTO [88]. miR-145 suppresses the proliferation of HCC by targeting YTHDF2 [89]. Similarly, miR-33a and miR-448 suppress the proliferation of NSCLC by targeting METTL3 and eIF3a [90, 91]. METTL3 is also downregulated by miR-600, which induces the apoptosis of lung cancer [92]. miR-141 suppresses the proliferation of pancreatic cancer by forming the miR-141/IGF2BP2/P13K/Akt axis [93]. Hepatitis B X-interacting protein (HBXIP) inhibits let-7 g expression to upregulate IGF2BP2, thus leading to the formation of a positive feedback loop of HBXIP/let-7 g/IGF2BP2/HBXIP to accelerate cell proliferation in breast cancer [94]. miR-497 partially reverses transforming growth factor beta 1 (TGFβ1)-induced epithelial-mesenchymal transition (EMT) and pulmonary fibroblast proliferation through inhibiting eIF3a in alveolar epithelial cells [95].

**Table 2:** NcRNAs modulate m^6^A regulators in cancers

Related non-coding RNA	m^6^A component	Cancer	Function	Role in cancer	Regulation	References
miR-33a	METTL3	NSCLC	Writers	Oncogene	Up-regulation	[90]
miR-600	METTL3	Lung cancer	Writers	Oncogene	Up-regulation	[92]
miRNA let-7g	METTL3	Breast cancer	Writers	Oncogene	Up-regulation	[94]
miR-1266	FTO	CRC	Erasers	Oncogene	Up-regulation	[88]
miR-145	YTHDF2	HCC	Readers	Oncogene	Up-regulation	[89]
miR-488	eIF3a	NSCLC	Readers	Oncogene	Up-regulation	[91]
miR-141	IGF2BP2	Pancreatic cancer	Readers	Oncogene	Up-regulation	[93]
lncRNA LINC00470	METTL3	GC	Writers	Oncogene	Up-regulation	[97]
lncRNA GATA3-AS	KIAA1429	HCC	Writers	Oncogene	Up-regulation	[104]
lncRNA GAS5-AS1	ALKBH5	Cervical cancer	Erasers	Anti-oncogene	Down-regulation	[102]
lncRNA GAS5	YTHDF3	CRC	Readers	Oncogene	Up-regulation	[103]
lncRNA LIN28B-AS1	IGF2BP1	LUAD	Readers	Oncogene	Up-regulation	[100]
lncRNA LINRIS	IGF2BP2	CRC	Readers	Oncogene	Up-regulation	[101]
lncRNA miR503HG	HNRNPA2B1	HCC	Readers	Oncogene	Up-regulation	[98]
lncRNA LINC01234	HNRNPA2B1	NSCLC	Readers	Oncogene	Up-regulation	[99]

*HCC* hepatocellular cancer, *GC* gastric cancer, *CRC* colorectal cancer, *LUAD* lung adenocarcinoma, *NSCLC* non-small cell lung cancer

lncRNAs also regulate m^6^A methylation in cancer. LncRNA derived from hepatocytes (lnc-HC) interacts with HNRNPA2B1 to inhibit cholesterol metabolism in hepatocytes [96]. Long intergenic non-protein coding RNA 470 (LINC00470) interacts with METTL3 to suppress the stability of phosphatase and tensin homolog (PTEN) to facilitate GC progression [97]. LncRNA miR503 host gene (miR503HG) also interacts with HNRNPA2B1 to promote its degradation through an ubiquitin-proteasome pathway in HCC [98]. Similarly, long intergenic non-protein coding RNA 1234 (LINC01234) interacts with HNRNPA2B1 to facilitate cell proliferation and inhibit cell apoptosis in NSCLC [99]. Lin-28 homolog B antisense RNA 1 (LIN28B-AS1) interacts with IGF2BP1 to promote the proliferation and metastasis of lung adenocarcinoma (LUAD) [100]. Long intergenic Noncoding RNA for IGF2BP2 Stability (LINRIS) promotes CRC proliferation by stabilizing IGF2BP2 [101]. The antisense RNA of growth arrest special 5 (GAS5-AS1) depends on ALKBH5 to suppresses the growth and metastasis of cervical cancer [102]. Growth arrest special 5 (GAS5) can suppress YAP-mediated YTHDF3 to restrain the proliferative behavior of CRC [103]. Antisense strand of the GATA binding protein 3 gene (GATA3-AS) enhances the interaction between KIAA1429 and GATA binding protein 3 (GATA3) pre-mRNA, leading to the formation of the GATA3-AS/KIAA1429/GATA3 axis in HCC [104].

## Clinical application of m^6^A methylation in cancer

m^6^A methylation serves as new biomarkers for diagnosis and prognosis in cancer. m^6^A regulators METTL3, YTHDC2 and HNRNPC are used to predict the prognosis in patients with HNSCC [105]. Upregulated METTL3/FTO or downregulated YTHDF2 and METTL14 can indicate a poor survival in GC, CRC, and HCC [48, 70, 106]. Low expression of METTL14 is associated with tumor differentiation, clinical stage, and microvascular invasion [48]. Low expression of ALKBH5 or FTO predicts an unfavorable marker in lung cancer and HCC [107, 108]. IGF2BP2 is considered as a prognostic marker in pancreatic cancer, esophagogastric junction adenocarcinoma and CRC [60, 109, 110].

m^6^A methylation also participates in drug resistance and cancer treatment. METTL3 stabilizes YAP and Rho GTPase activating protein 5 (ARHGAP5) to induce cisplatin resistance in NSCLC and in GC [69, 111]. HNRNPA2B1 is overexpressed in tamoxifen-resistant breast cancer and reduces 4-hydroxytamoxifen sensitivity [112]. In addition to METTL3 and METTL14, FTO and YTHDF2 are overexpressed in AML [26, 27, 113, 114]. A recent study shows that FTO inhibitor (FB23) and its derivative (FB23–2) promote myeloid differentiation and apoptosis in AML by targeting FTO [115]. m^6^A methylation is also involved in estimating tumor microenvironment and TME infiltration characterization so as to provide insights into an effective immunotherapy for cancer [116]. YTHDF2 is correlated with inflammation infiltration, vascular reconstruction and distant metastasis and predicts a poor prognosis in HCC [117].

In summary, the role of m^6^A modification in clinical application has been widely validated. As for the core members of m^6^A methylation, METTL3/14 exert their functions in many biological processes. METTL3/14 can be regarded as the most important and promising m^6^A regulator and arouse our attention about their modifications on ncRNAs and the clinical application in cancer diagnosis.

## Conclusions and perspectives

Accumulating studies have been focused on how m^6^A methylation modifies the stability, splicing and translation of ncRNAs or ncRNAs regulate m^6^A regulators in cancer. The interaction between m^6^A methylation and ncRNAs can impact the different life activities including cancer cell proliferation, invasion and metastasis. As for the clinical application of m^6^A methylation, they can be regarded as the potential targets for cancer diagnosis, prognosis and treatment. The latest findings show that lncRNA long intergenic non-protein coding RNA 266–1 (LINC00266–1) interacts with IGF2BP1 by encoding a 71-amino acid peptide, named RNA-binding regulatory peptide, thereby promoting tumorigenesis [118]. However, the specific binding sites between m^6^A methylation and ncRNAs need be further investigated.

## Data Availability

All data generated or analyzed during this study are included in this published article and its additional files.

## Abbreviations

3′-UTR: 3′ -untranslated region
ALKBH5: alkB homologue 5
AML: Acute myeloid leukemia
ARHGAP5: Rho GTPase activating protein 5
circRNAs: circular RNAs
circNSUN2: circular RNA NOP2/Sun RNA methyltransferase 2
circPVRL3: circular RNA poliovirus receptor-related 3
circ-ZNF609: circular RNA zinc finger protein 609
CRC: Colorectal cancer
CSC: Cigarette smoke condensate
DANCR: Differentiation antagonizing non-protein coding RNA
DGCR8: DiGeorge syndrome critical region 8
eIF3: eukaryotic translation initiation factor 3
EMT: Epithelial-mesenchymal transition
ESCC: Esophageal squamous cell carcinoma
FAK: Focal adhesion kinase
FGFR3: Fibroblast growth factor receptor 3
FAM225A: Family with sequence similarity 225 member A
FTO: Fat mass and obesity-associated protein
FOXM1: Forkhead box M1
FOXM1-AS: antisense to forkhead box M1
GAS5: Growth arrest special 5
GAS5-AS1: the antisense RNA of GAS5
GATA3: GATA binding protein 3
GATA3-AS: Antisense strand of the GATA binding protein 3 gene
GC: Gastric cancer
GSCs: Glioblastoma stem-like cells
HBXIP: Hepatitis B X-interacting protein
HCC: Hepatocellular carcinoma
HIF-1α: Hypoxia-inducible factor 1-alpha
HMGA2: High mobility group AT-hook 2
HNRNP: Heterogeneous nuclear ribonucleo protein
HNSCC: Head and neck squamous cell carcinoma
IGF2BP1/2/3: Insulin-like growth factor-2 mRNA-binding proteins 1/2/3
ITGB3: Integrin β3
LINC00266–1: Long intergenic non-protein coding RNA 266–1
LINC00278: Long intergenic non-protein coding RNA 278
LINC00470: Long intergenic non-protein coding RNA 470
LINC00958: Long non-coding RNA 00958
LINC01234: Long intergenic non-protein coding RNA 1234
LIN28B-AS1: Lin-28 homolog B antisense RNA 1
LINRIS: Long intergenic Noncoding RNA for IGF2BP2 Stability
LNCAROD: lncRNA activating regulator of DKK1
lnc-HC: LncRNA derived from hepatocytes
lncRNAs: Long non-coding RNAs
LUAD: Lung adenocarcinoma
m^6^A: N6-methyladenosine
m^6^A-seq: N6-methyladenosine-sequensing
MALAT1: Metastasis-associated lung adenocarcinoma transcript-1
MEFs: Mouse embryonic fibroblasts
METTL3/14/16: Methyltransferase-like 3/14/16
miR503HG: miR503 host gene
miRNAs: Micro RNAs
mRNA: Messenger RNA
ncRNAs: Noncoding RNAs
NEAT1: Nuclear paraspeckle assembly transcript 1
NSCLC: Non-small cell lung cancer
PDAC: Pancreatic ductal adenocarcinoma
PTEN: Phosphatase and tensin homolog
RBM15/15B: RNA-binding motif protein 15/15B
Ribo-circRNAs: Ribosomes-circRNAs
RP11: RP11–138 J23.1
SAM: S-adenosylmethionine
STING: Stimulator of interferon genes
TGFβ1: Transforming growth factor beta 1
WTAP: Wilms tumor 1-associated protein
XIST: X-inactive specific transcript
YAP: Yes associated protein
YTH: YT521-B homology
YTHDC1/2: YTH domain-containing proteins 1/2
YTHDF1/2/3: YTH domain family 1/2/3
